# Museum ‘dark data’ show variable impacts on deep-time biogeographic and evolutionary history

**DOI:** 10.1098/rspb.2024.2481

**Published:** 2025-02-26

**Authors:** Christopher D. Dean, Jeffrey R. Thompson

**Affiliations:** ^1^ Department of Earth Sciences, University College London, London, UK; ^2^ School of Biological Sciences, University of Southampton, Southampton, UK; ^3^ School of Ocean and Earth Science, University of Southampton, Southampton, UK

**Keywords:** Paleobiology Database, fossil record bias, big data, curation, collections

## Abstract

The age of digitally accessible datasets has transformed palaeontology, enabling previously impossible macroevolutionary insights. However, a substantial reservoir of generally inaccessible ‘dark data’ resides within museum collections, which may alter our understanding of ancient groups and their ecological and evolutionary history. We demonstrate how the addition of data held exclusively in museums impacts our macroevolutionary understanding of an entire taxonomic group, using a dataset of Palaeozoic echinoids containing the majority of museum occurrences for the clade. We find that museum ‘dark data’ shows clear differences in composition compared to data available in the published literature and strongly impacts biogeographic patterns, increasing the average geographic range size of taxa by 35%. Global model results assessing drivers of diversity are also significantly affected by the addition of museum-only data. Conversely, ‘dark data’ have a more limited impact on the temporal ranges of taxa or estimates of overall diversity and are impacted by similar socio-geographic biases as the published record. These findings show that unpublished museum data are necessary to obtain a complete understanding of macroevolutionary patterns in deep-time, illustrating the importance of the collection, curation, digitization and continued care of ‘dark data’ in the age of ‘Big Data’ in palaeobiology.

## 1. Introduction

The fossil record is unique among the natural sciences in providing a chronicle of life and evolution on Earth through both space and deep-time, allowing scientists to transcend the temporal limitations of contemporary ecological datasets. Assessing how organisms have historically responded to major environmental changes across local, regional and global scales provides critical information on how species will respond to our current biodiversity crisis and has consequently been a central goal of palaeontology over the past half-century [1,2]. This objective has been made increasingly possible through leveraging digitally available aggregated databases of fossil occurrences for macro-scale analyses. Since the origins of modern ‘quantitative palaeontology’ in the 1970s [3], large compilations of fossil data gathered from the published literature have become an increasingly important resource in the palaeontological toolkit, and online databases such as the Paleobiology Database (PBDB) [4], Neotoma [5] and Triton [6] have increased the accessibility of examining progressively broader scale questions regarding macroevolutionary processes and Phanerozoic-scale biodiversity patterns [2,7,8].

However, a significant barrier to effectively utilizing such resources is that these large palaeontological datasets are far from a complete account of the available fossil record. Aside from issues caused by incomplete taxonomic, spatial and temporal sampling [9–13], the knowledge documented and published in the scientific literature that comprises these databases is only a fraction of the available palaeontological data accessioned within museums, which additionally consists of unpublished specimens [14–16]. Recent work by Marshall *et al*. [16] has shown that museums across the Western coast of the USA contained 23 times more marine localities than were recorded in the PBDB at the time. Extrapolating this figure provides an estimate of as little as 3−4% of total known localities around the world as being accounted for within the PBDB, suggesting that a significant amount of recorded information about the fossil record remains currently inaccessible. This supports prior work which found that the published record underestimated diversity of various invertebrate groups in the late Cenomanian of the North American Western Interior Seaway by a factor of between 3 and 5 [14]. These studies have been restricted in both geographic and taxonomic scope, and the impact of this ‘dark data’ [17] on our understanding of the evolutionary history of entire taxonomic groups, a frequent analytical unit of palaeobiology, is yet to be examined. Crucially, this limits the questions we can reasonably ask about the fossil record. It is currently unclear whether the addition of data held exclusively in museums will greatly change perceived spatial or temporal patterns in ancient taxonomic groups, or impact knowledge regarding the drivers of biodiversity, biogeography, environmental associations, and potential geological and anthropogenic biases through deep time. Understanding whether the addition of ‘dark data’ to publicly accessible fossil occurrence datasets significantly changes interpretations about the fossil record is therefore a critical question in macroevolution and macroecology more broadly, particularly as we enter an age of palaeontological ‘Big Data’ [15].

Herein, we examine the potential impacts of museum ‘dark data’ by comparing and contrasting datasets containing Palaeozoic occurrences of a skeletonized marine invertebrate group (class Echinoidea). These datasets are produced from (i) the available published literature, which also includes data from (ii) the PBDB (http://paleobiodb.org) and (iii) a taxonomically standardized record of unpublished museum specimens, gathered from 33 museum collections worldwide. Together, they contain the majority of known Palaeozoic echinoids residing in museums and span ~200 million years of evolutionary history. We use these datasets to produce estimates of typical biological patterns used within macroecological research, such as diversity, occupancy, geographic range size and temporal range, as well as carry out model-based evolutionary analyses, and compare these results against analyses carried out using all currently available data. Our goal is to discern the impact of museum ‘dark data’ on the understanding of macroevolutionary and ecological patterns in the fossil record, and on our interpretations of the drivers of these patterns.

## 2. Methods

### (a) Occurrence datasets and data preparation

To compare the relative differences between museum ‘dark data’ (unpublished museum occurrences), the PBDB and the broader published record, we constructed a total of four datasets: (i) the ‘dark data’ dataset (dataset A), containing specimens reported in museums only (and therefore not present in the published record); (ii) the published dataset (dataset B), containing specimens described in the published record; (iii) the PBDB dataset (dataset C), a subset of the published dataset containing only the occurrences that are present in the PBDB; and (iv) the combined dataset (dataset D), which contains the total unique information from all the previous datasets (essentially, dataset A plus dataset B). This allowed us to compare patterns between ‘dark data’, the PBDB and published records, as well as assess their relative contribution to the complete record of data (full details of datasets can be found in electronic supplementary material, table S1). It should be noted that as a community project, the PBDB is not comprehensive and does not contain the full published record of fossils for most taxonomic groups and is instead a reflection of research interests and available resources. We downloaded a comprehensive genus-level dataset of Palaeozoic (538.8−251.902 Ma) echinoid body-fossil occurrences from the PBDB on 21/09/2023 using the group name ‘Echinoidea’ and time intervals ‘Cambrian’ to ‘Permian’, which was then vetted for taxonomic and stratigraphic consistency. We then expanded upon the museum-based dataset of Carboniferous echinoid specimens published by Thompson & Bottjer [18] to include specimens from the entirety of the Palaeozoic, additionally adding georeferencing information; specimens without precise geographic co-ordinates were georeferenced by locating text-based locality descriptions on Google Maps (available from http://google.com/maps). This dataset was also used by Thompson *et al*. [19], which contains full information regarding its construction. Taxonomic identification across all datasets was carried out by one individual (J.R.T.) to ensure consistency in approach and remove a potential source of bias between datasets [20]. This dataset was compiled from visits to 33 institutional collections across the United States and Europe and includes taxonomic identification and associated metadata (taphonomic grade [see electronic supplementary material, figure S2 and Thompson *et al*. [19] for details], lithology, and grainsize) at a specimen level (see electronic supplementary material, table S2 for full list of institutions). Museums were chosen for visitation based on publicly available knowledge of them containing Palaeozoic echinoids, including published literature, online collections databases and *a priori* contact with collections managers/curators. While Palaeozoic echinoids have been found globally, their sampling and representation in institutions outside of North America and Europe is limited. These factors are reflected in our dataset; potential impacts of this can be found in the Discussion. To enable comparison to the occurrence-based PBDB dataset, duplicate appearances of taxonomic occurrences from the same collections were removed. The finalized total dataset (dataset D) consists of 3447 total specimens and 2223 occurrences; the ‘dark data’ dataset (dataset A), Published record (dataset B) and PBDB (dataset C) datasets contain 976, 1247 and 590 occurrences respectively [21]. Occurrences from resulting datasets were assigned to stage and sub-period-level time bins using the ‘majority’ methodology of the bin_time() function from the R package ‘palaeoverse’ [22]; the latter time scale splits the Carboniferous into the Mississippian and Pennsylvanian, due to the abundance of echinoid specimens recovered from this interval [23]. We also downloaded additional datasets of echinoderms and Palaeozoic invertebrates from the PBDB for correlation and occupancy comparisons. The full list of the parameters used for the PBDB download and the raw datasets themselves can be found in electronic supplementary material 2.

**Table 1.** Table showing the best fitting GLS models assessing genus-level diversity of echinoids for the complete dataset and subsets at data, at either North American or global scale. Carb: carbonate units; hardie: Ca/Mg ratio from Stanley & Hardie [35]; mean_sl: mean sea level; colls: number of echinodermata collections; wilkinson: Ca/Mg ratio from Wilkinson & Algeo [36]; df: degrees of freedom; loglik: log likelihood; AICc: Akaike information criterion, corrected for small sample sizes.

model	data	best-fitting model for dataset	df	loglik	AICc
North America only	all	(intercept)+carb + hardie	5	4.002	4.496
North America only	dark data	(intercept)	3	2.294	3.259
North America only	PBDB	(intercept)+carb + hardie	5	20.547	−28.367
North America only	published	(intercept)+carb + hardie+mean_sl	6	6.985	1.682
global	all	(intercept)+colls	4	2.505	4.472
global	dark data	(intercept)+colls	4	2.058	5.550
global	PBDB	(intercept)+colls + hardie+mean_sl+wilkinson	7	18.301	−17.935
global	published	(intercept)+colls + hardie+mean_sl	6	3.745	7.870

We worked from the base hypothesis that patterns and inferences regarding the evolutionary history of Palaeozoic echinoids would be the same across datasets. To test this hypothesis, we carried out comparative, geographic and temporal, and model-based approaches. Note that we focused on comparing the spread of data between datasets, rather than examining or comparing potential errors in the values of the datasets (e.g. originally misidentified taxa). All analyses were carried out in R version 4.2.2 [24], and scripts to run all analyses are available in the electronic supplementary material.

### (b) Comparative tests

To establish comparisons between the datasets, we assessed differences in the proportions of taxonomic ranks, lithology (carbonate or siliciclastic host rock), grain size (fine grained or coarse grained) and taphonomic grade (1–5). These values were assigned using the protocol in Thompson *et al*. [19], where further details can be found. Proportions of metadata from each dataset were compared and tested using the chi-squared test, Kruskal–Wallis test and Wilcoxon rank-sum test. Mosaic plots were produced using the mosaic() function of the package ‘vcd’ [25]. To assess relationships between the locations of fossil collection and repository for the different datasets, we followed the approach of Raja *et al*. [12], adapting their R scripts.

### (c) Geographic and temporal approaches

We calculated measures of occupancy, latitudinal range and geographic range size of taxa for each dataset through time to establish whether museum-only data were affecting biogeographic patterns. We used the ‘PALEOMAP’ rotation model through the palaeorotate() function of the package ‘palaeoverse’ [22] to obtain palaeocoordinates. Global occupancy of grid cells at a resolution of 1° was calculated for all stages combined and at individual stage levels for all datasets, as well as for total Palaeozoic echinoderms and invertebrates to act as a baseline for the approximate spatial spread of data. Geographic ranges for individual genera were calculated for each stage with each dataset using a convex hull encompassing all palaeocoordinates of occurrences for that genera; we then calculated the difference between the range from the complete dataset and each sub-dataset for each taxon per stage, as well as the area added with the inclusion of museum data (figure 2A). This same approach was used for assessing differences in latitudinal range through time (figure 3A). As choice of palaeorotation model can impact geographic measures, particularly in the Palaeozoic [26], we also generated geographic range results using the ‘MERDITH2021’ palaeorotation model to test for consistency. The mean and median latitudinal ranges were also calculated for each subset of data and compared to the complete dataset to assess if there had been a shift with the addition of data. Initial measures of biogeography (occupancy, latitudinal range and geographic range) were calculated using the ‘occ’, ‘lat’ and ‘con’ methods of the tax_range_space() function from the package ‘palaeoverse’ [22]. We also calculated temporal range at the family, genus and species level for each dataset. We limited taxa to those found in a maximum of two bins to ensure that temporal uncertainty was not influencing results. To compare data, we established overlap with the total range, as well as the percentage of time added when including museum data. Temporal ranges were calculated using the tax_range_time() function of ‘palaeoverse’ [22].

**Figure 1.**
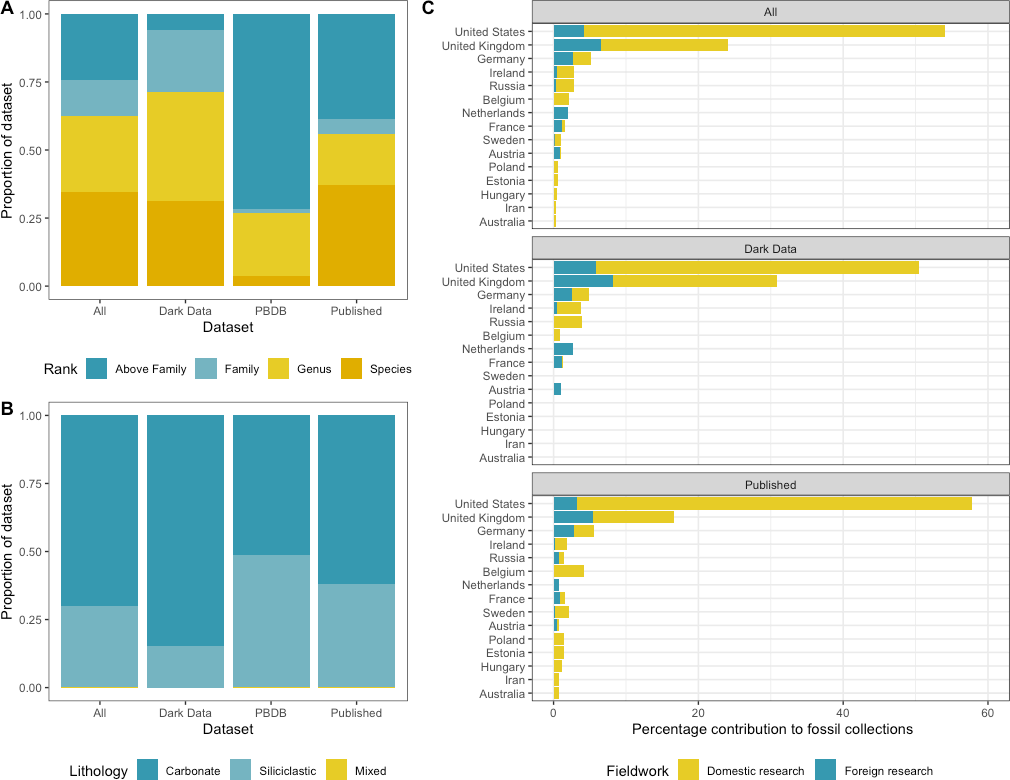
Figure showing comparisons of various metadata between datasets. (A) Comparison of datasets broken down by taxonomic rank, as proportion of total. (B) Comparison of datasets broken down by associated lithology, as proportion of total. (C) Percentage contribution of the top 15 countries to accessioned fossil occurrences, shown for subsets of total data. The colour of each bar represents the total contribution from either domestic (fossils found and accessioned in a museum in the same country) or foreign (fossils found and accessioned in separate countries) research.

**Figure 2.**
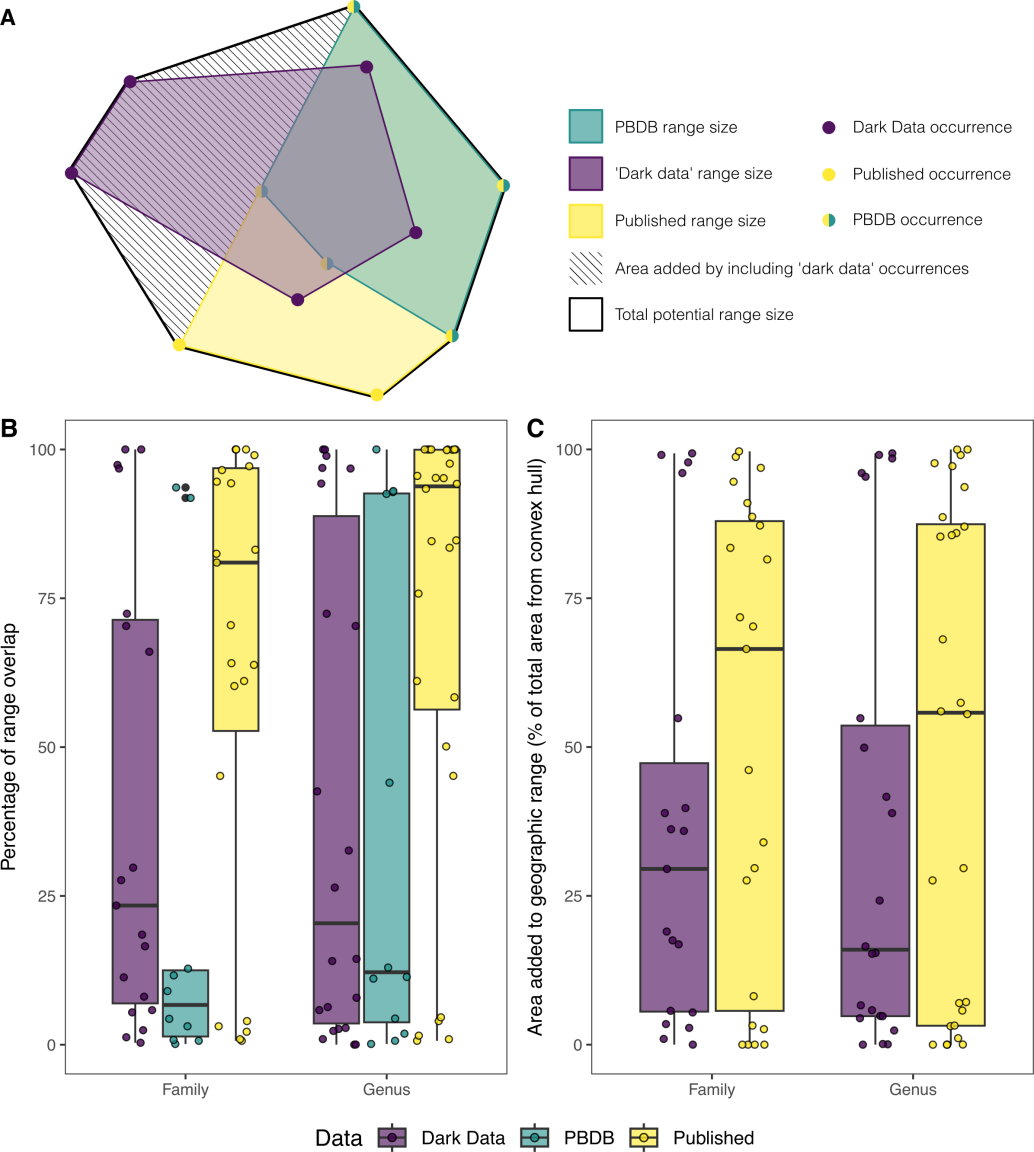
Comparisons of geographic range between datasets. (A) Schematic diagram showing the breakdown of geographic range sizes between datasets for a single taxon, including the hypothetical area added when ‘dark data’ are included alongside occurrences from the published record. Note how additional area also includes area outside of the range of ‘dark data’ occurrences, but within the overall convex hull. (B) Box plots showing the percentage of geographic range size overlap between the complete dataset and subsets for individual taxa through time, at genus and family taxonomic ranks. In (A), this would be the percentage that each dataset makes up of the total potential range size (e.g. ‘dark data’ might make up 50% of the total potential range size for the taxon exhibited in (A)). (C) Graph showing how much area is added to the total geographic range size for individual taxa when a specific subset of data is added, expressed as percentage of the total geographic range size, shown for genus and family taxonomic ranks. In (A), this would be the percentage that the hashed area makes up of the total potential range size for each dataset.

### (d) Diversity analysis

We calculated both raw and subsampled estimates of diversity through time for each dataset to establish whether the inclusion of ‘dark data’ substantially changed macroevolutionary analyses. Both genus-level raw diversity and the number of collections for each stage were calculated using the divDyn() function of the ‘divDyn’ package [27]. We then carried out coverage-based rarefaction, a commonly used technique in palaeobiological analyses that aims to achieve a more fair estimate of diversity through estimating the coverage of samples [28]. This was carried out using the estimateD() function of the package iNEXT [29], using the ‘incidence_freq’ datatype and ‘coverage’ as the comparison base. Correlations between datasets were tested after log transformation using the Spearman’s rank correlation test, with the Benjamini–Hochberg correction for large sample sizes applied to reduce the chance of acquiring type I statistical errors [30].

### (e) Standardized relative affinity

To understand the impact of adding ‘dark data’ to studies that assess the attributes of individual taxa, we calculated the standardized relative affinity (SRA) of echinoids for particular substrates (carbonate or siliciclastic), following the protocol outlined and the R code provided in Thompson & Bottjer [18], originally adapted from Miller & Connolly [31]. Due to low sample sizes for other families, we only established the SRA through time for the Archaeocidaridae, Lepidesthidae, Lepidocentridae, Palaechinidae and Proterocidaridae. For interpretation, a positive SRA indicates the given family has a relative affinity for carbonate substrates, whereas a negative value indicates a disinclination.

### (f) Linear modelling

Finally, we carried out linear modelling to test whether different datasets ended up with comparable covariates included within their respective best-fitting models. By comparing models run with each subset of the complete dataset (datasets A, B or C) to the complete dataset (dataset D, which we regard as our most complete picture of the record of Palaeozoic echinoids), we aim to see what commonalities we observe among the covariates in the respective best-fitting models, and whether interpretations of diversity drivers change when ‘dark data’ are added. We used raw genus-level diversity as our response variable, as small sample sizes prohibited the use of coverage-based rarefaction estimates, which were taken from the diversity analyses described above. We next obtained covariate time series data which had the potential to impact echinoid diversity, including sea level, magnesium/calcium ratios, proxies for sampling effort (Good’s *u* and echinoderm collections downloaded from the PBDB) and the abundance of carbonate and siliciclastic rocks in the North American continent. Full details on how these covariates were obtained and processed can be found in the electronic supplementary material. All covariates were log normalized prior to analysis. We used generalized least squares (GLS) multiple regression models to simultaneously examine relationships between diversity and multiple time series covariates. GLS models can fit autoregressive models to reduce the chance of results being impacted by serial correlation; here we use the first-order autoregressive correlation model, which seeks autocorrelation at up to one lag in either direction. Best-fitting models were identified by comparing the outputs of different combinations of explanatory variables using Akaiki’s information criterion corrected for small sample sizes (AICc) [32]; lower AICc values indicate better-fitting models. Modelling was carried out using the gls() function of the ‘nlme’ package [33]. Model selection was carried out using the dredge() function of the package ‘MuMIn’ [34].

## 3. Results

### (a) Compositional differences in datasets

To characterize potential inherent differences across sources of data, we first compared the lithological, taxonomic and taphonomic breakdown of occurrences within our three datasets. We observe large differences in overall composition between ‘dark data’ occurrences and those in the published record (figure 1; electronic supplementary material, figure S1). ‘Dark data’ occurrences are predominantly associated with carbonate lithologies (~85% of specimens), in contrast to the published record where siliciclastic-associated occurrences are far more common (~38% of specimens; figure 1B). ‘Dark data’ also show a higher overall proportion of coarse-grained sediment association than any other dataset (~26%). When broken down by taxonomic rank (figure 1A), the ‘dark data’ dataset contains proportionally more occurrences at the genus level than any other dataset (~40%), and the lowest proportion of occurrences classified to above family level (~6%). We also compared the taphonomic composition of fossil specimens (rather than occurrences). A statistically significant difference (X^2^ = 87.533, *p* = < 2.2e^−16^) is observed between the preservation of specimens found solely in museums versus the broader literature, with museums containing proportionally more specimens with poorer preservation scores (electronic supplementary material, figure S2).

**Figure 3.**
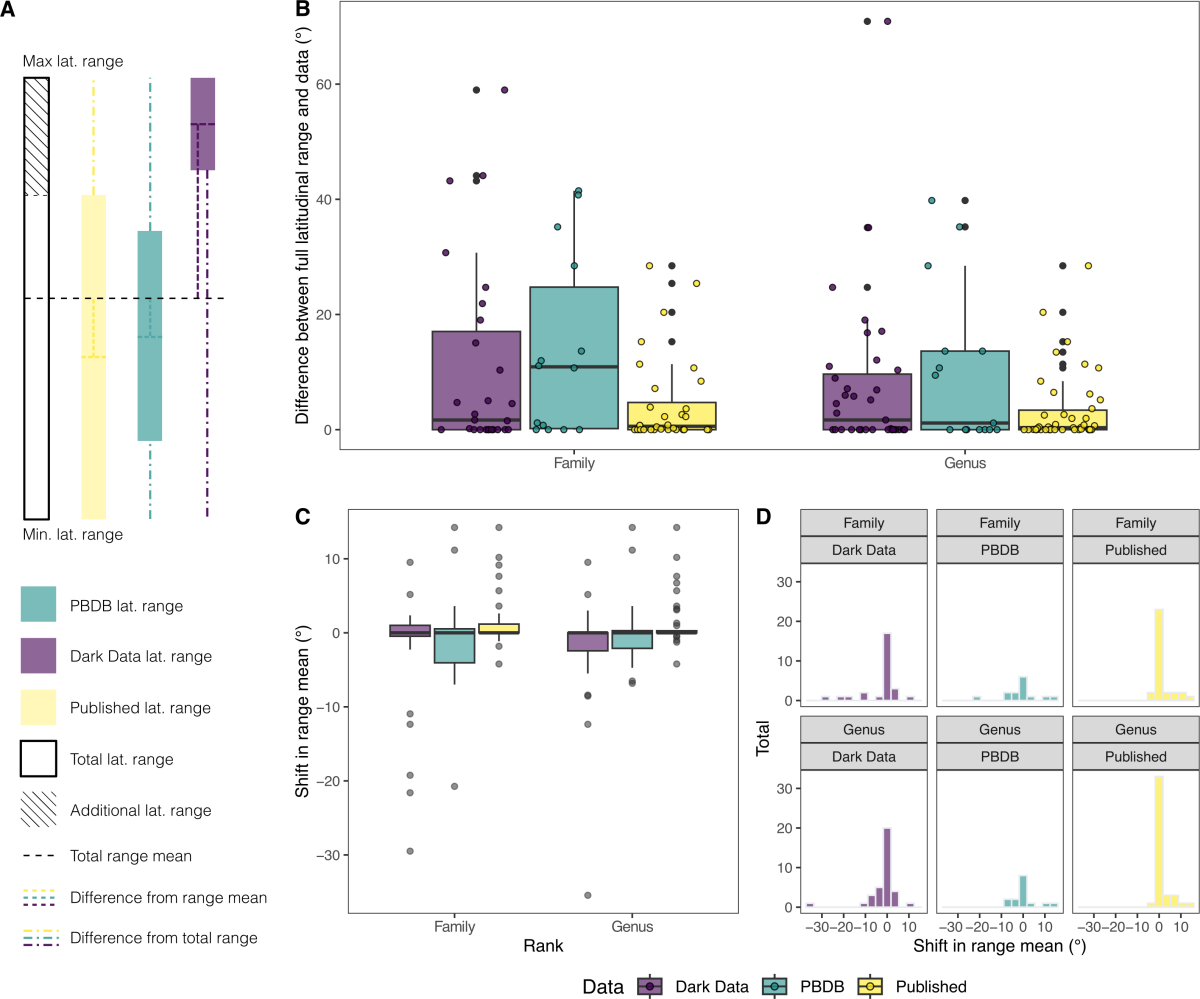
Comparisons of latitudinal range between datasets. (A) Schematic diagram showing the breakdown of latitudinal ranges between datasets for a single taxon, as well as the hypothetical additional latitudinal range when ‘dark data’ specimens are added alongside specimens from the published record and associated differences from range mean and the total range. (B) Box plots showing the difference in latitudinal degrees between latitudinal range of the complete dataset and individual subsets of data for taxa through time, at both genus and family ranks. (C) Box plots showing the shift in mean of the latitudinal range between the complete dataset and individual subsets for individual taxa through time, at both genus and family ranks. (D) Histograms showing the same information as displayed in (C).

Differences between datasets also extend to collection practices. Palaeozoic echinoids have been found in 41 countries, from 6 continents, with the published record having the largest spread (39 countries total); in comparison, ‘dark data’ occurrences come from a total of 18 countries (electronic supplementary material, figure S3). All suitable datasets report the same top three holders of Palaeozoic echinoid collections (United States of America, United Kingdom and Germany; figure 1C) which make up a total of ~86%, ~80% and ~83% of collections in the ‘dark data’, published record and complete datasets, respectively.

### (b) Biogeographic and temporal ranges

Including ‘dark data’ occurrences causes a variety of changes to biogeographic patterns. When assessing global occupancy (the proportion of 1° × 1° grid cells that contain a fossil occurrence), the spatial spread of the Palaeozoic echinoid record is increased by ~29% through the addition of museum ‘dark data’. Occurrences from the combined dataset cover 305 grid cells across the globe, equating to ~32% of grid cells featuring Echinodermata occurrences and ~9% of total Palaeozoic invertebrate coverage from the PBDB. In turn, ~77% (236) of the total covered grid cells uniquely came from the broader published record, and ~23% (69) came from ‘dark data’. When assessed through time (electronic supplementary material, figure S4), the published record shows the highest mean occupancy and number of uniquely occupied grid cells (~8.5, 6.11 cells, respectively) in comparison to ‘dark data’ (6.8, 4.32) or PBDB (4.4, 0) datasets. However, comparing the ratio between uniquely contributed grid cells from ‘dark data’ and published record datasets reveals a shift during the Carboniferous where unpublished museum collections provide a greater amount of unique data (electronic supplementary material, figure S4B).

We next calculated the geographic range size for individual taxa using a convex hull at the generic and family rank through time within each dataset and compared their respective sizes to the total geographic range of those taxa (figure 2A). Geographic ranges for genera calculated using the ‘dark data’ dataset showed mean and median area overlaps with the total geographic range of each genus of 40.4 and 20.4%, respectively (figure 2B). When looking at the total geographic area added by different datasets, including ‘dark data’ added an extra 35.2% area on average to the total geographic range of genera, and 36.8% to family ranges (figure 2C; electronic supplementary material, table S3). However, the Kruskal–Wallis test reported no statistically significant difference between the overall geographic range sizes in any dataset (X^2^ = 2.98, df = 2, *p* = 0.225, eff. size = 0.0166 [small]). These results are consistent across plate models used for palaeorotation (electronic supplementary material, table S3).

To compare latitudinal ranges between datasets, we examined the pairwise difference between the ranges of taxa from the complete dataset and individual subsets per stage (figure 3A). ‘Dark data’ genera show reasonably close similarity with the overall dataset, with a mean difference of 7.61° and a median difference of 1.68° (figure 3B), corresponding to a mean overlap of 67.6% and a median overlap of 89% (electronic supplementary material, table S4). ‘Dark data’ show slight northern skew in the mean of latitudinal ranges compared to the overall dataset, whereas published occurrences and the PBDB show a slight skew of range means to the south (figure 3C,D). However, Kruskal–Wallis and Wilcox tests showed no statistically significant difference between groups for either latitudinal range differences or overall range sizes. Adding ‘dark data’ occurrences to published data results in the latitudinal range of genera increasing by an average of ~28.9% (~4.1° in latitude).

**Figure 4.**
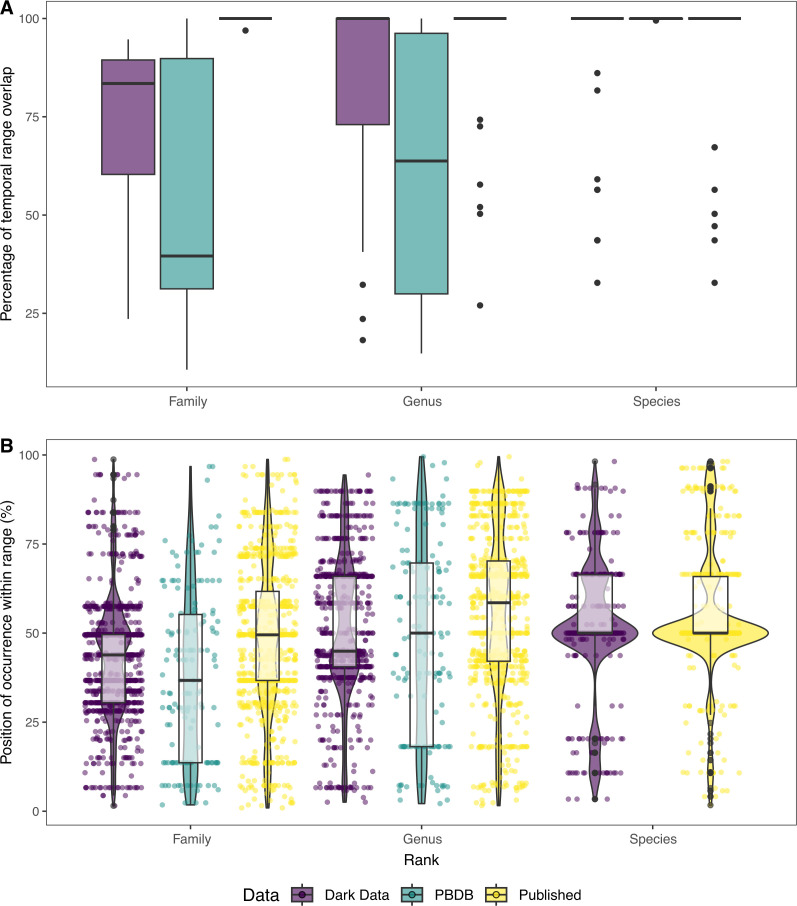
Figure showing comparisons of temporal range between datasets. (A) Box plots showing the overlap in temporal range between the complete dataset and individual subsets of data for individual taxa through time expressed as percentage of total, at species, genus and family taxonomic ranks. (B) Violin plots showing the distribution of individual occurrences within the total temporal range for their taxon across the various datasets, at species, genus and family taxonomic ranks.

We also assessed the difference in temporal ranges of taxa between the complete dataset and subsets at different taxonomic ranks (figure 4). The published record shows the closest similarity to the temporal ranges of the complete dataset, on average covering ~98% of the total temporal range (figure 4A). ‘Dark data’ ranges are relatively accurate at the species (~91.8% temporal coverage) and genus levels (~80.9%), but are slightly worse at the family (~73.1%) rank. Adding ‘dark data’ occurrences results in a mean temporal range increase of ~5.4% for species, ~8.9% for genera, and 0.4% for families. When comparing the distribution of individual occurrences in the temporal range of their respective taxonomic units (i.e. at what position throughout the total temporal range of a taxonomic unit was an individual occurrence found), the published record shows a more even distribution than that of ‘dark data’ for family and genus ranges, but little difference at the species rank (figure 4B).

### (c) Impacts on analyses of macroevolutionary patterns

The inclusion of ‘dark data’ is also shown to have variable impacts on the outcomes of common macroevolutionary analyses and interpretations of the echinoid fossil record. When comparing estimates of raw diversity through time (electronic supplementary material, figure S5), the published record and ‘dark data’ datasets show the closest similarity to overall diversity compiled from all datasets, showing a significant peak in the early Carboniferous; this is supported by statistically significant Spearman’s rank correlation coefficients (‘dark data’: r_s_ = 0.81, *p* = 4.77e^−8^; published: r_s_ = 0.94, *p* = 1.01e^−15^; electronic supplementary material, table S6). The record from the PBDB shows a flatter profile, although it still shows a strong and statistically significant correlation with the overall pattern of diversity (r_s_ = 0.528, *p* = 0.00187).

**Figure 5.**
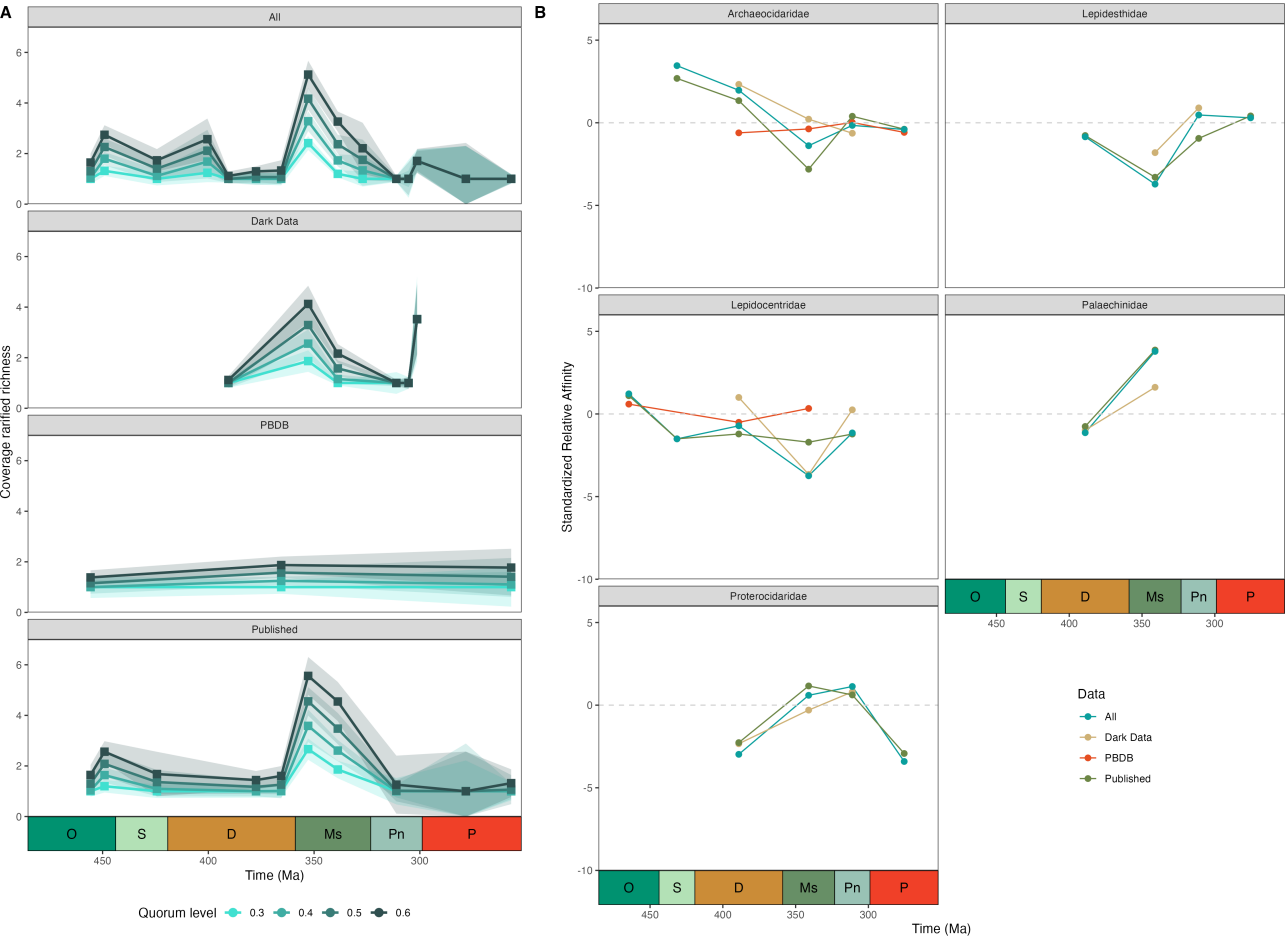
Figure showing comparisons of different time series analyses between datasets. (A) Time series showing coverage-based rarefaction diversity for the complete dataset, as well as individual subsets of data, at various quorum levels. (B) Time series of standardized relative affinities (SRA) showing substrate affinity for five families of echinoids during the Palaeozoic, calculated using the complete dataset and subsets of data at the series level. A positive SRA indicates affinity for carbonates, a negative SRA indicates affinity for clastics and an SRA of 0 indicates mixed or no affinity.

When comparing diversity estimates produced using coverage-based rarefaction (figure 5A), both ‘dark data’ and published datasets distinguish key features observed within the complete dataset, including a mid-Devonian low, an early Mississippian peak, and a decline into the Pennsylvanian. The museum record also shows a large diversity increase prior to the Carboniferous/Permian boundary which is not observed within the published record; this appears as a relatively small fluctuation in the complete dataset. Due to low occurrences, the PBDB dataset was only able to resolve three points throughout the Palaeozoic and thus cannot be accurately compared. Trajectories of sediment affinity (SRA) through time for different families of echinoids are broadly consistent across datasets (figure 5B); only the PBDB shows a substantial deviation for several datapoints.

Finally, we compared GLS model outputs of each dataset to establish the impact of varying data sources on understanding the potential drivers of echinoid diversity through time. When restricted to occurrences from North America (table 1; electronic supplementary material, table S7), the best-fitting model for the complete dataset (dataset D) included carbonate area and Mg/Ca ratio as statistically significant covariates, closely matching covariates included in the best-fitting models for the published record (dataset B) and PBDB datasets (dataset C). However, when widened to including global occurrences (table 1; electronic supplementary material, table S8), ‘dark data’ appears to have a significant impact on model outputs. Only the number of collections was included as a statistically significant covariate in the best-fitting model using the complete dataset, which matched the best-fitting model of the ‘dark data’ dataset (dataset A); datasets for the published record and PBDB however included additional statistically significant covariates (mean sea level, as well as Mg/Ca ratios) within their top models.

## 4. Discussion

When comparing specimen-based datasets, we observe that museum ‘dark data’ shows clear differences in composition across a variety of metadata compared to specimens featured in the PBDB or the broader published literature. Museum specimens show statistically significant differences in the proportions of lithology, grainsize, taxonomic rank and specimen quality/completeness compared to data collected from the published record or from the PBDB. These differences are likely to at least partially reflect contrast in overall preservation quality between datasets. Echinoids are relatively rare components of Palaeozoic ecosystems in comparison to the Mesozoic or Cenozoic [18,37] and also exhibit large variation in preservation state during the Palaeozoic due to the lack of microstructural interlocking in their multi-element skeleton, leading to disarticulation following death [19,38]. This combination of factors is likely to have influenced relative collecting practises in the field; instead of preferential collection based on preservation quality as can occur with abundant invertebrate remains [39,40], incomplete specimens of Palaeozoic echinoids would still be recovered due to their relative rarity, similar to the collection of vertebrate material from the fossil record. These specimens are also less likely to be included within the published record in comparison to those with higher taphonomic fidelity. The impact of museum specimens showing lower quality of preservation could also explain deviations in the other associated metadata; siliciclastic and fine-grained sediments are more likely to be associated with complete specimens in comparison to carbonate and coarse-grained [19,41,42], which show increased proportions in the museum specimens dataset.

All datasets examined here exhibit distinct spatial heterogeneity in terms of both where fossils were recovered and where they are reposited in museums, with a strong bias towards Europe and North America. The United States, United Kingdom and Germany are found to contain the majority of specimens housed in museums; these are also the top three countries previously found to have the largest overall contributions to palaeontological data in the PBDB [12]. Equally, Europe and North America account for an average of ~99% of fossil occurrences in museums, extremely similar to the ~97% of fossil occurrence data contributed by authors primarily based in those continents [12]. This might in part be due to past and present methods of data collection, and historical biases in collection practises. Palaeozoic echinoids are renown for having a poorly sampled record outside of Europe and North America [18], and as such these continents were the focus for historic and present data collection. While this dataset is currently unprecedented in its coverage, it is highly likely that additional Palaeozoic echinoid occurrences are present in museums that were not visited by the relevant author (J.R.T.), particularly in the global South. Researchers planning museum visits are likely to choose locations based on where they know specimens for their group of interest are present, which will predominantly be based on the published record. This creates a self-perpetuating cycle, where museums containing only unpublished specimens for a group will be ignored by visiting researchers, entrenching preconceived notions of where specimens are held. Such an issue is further exacerbated by museums which may be lacking online services or presence entirely. We acknowledge that we have also played into this pattern through selecting museums to visit based on these criteria, but hope that this work further exemplifies the need for global digitization efforts and additional support for local scientists to ensure accuracy in our knowledge of where fossil materials are held. Considering these points, it is easy to read the available fossil record of Palaeozoic echinoids as one that is at least partially a product of colonial legacy. However, unlike results from the wider PBDB, Palaeozoic echinoids have a significantly higher proportion of domestic research, indicating a relative lack of ‘parachute science’ [43]. One potential cause is that Palaeozoic echinoids, despite their relative rarity, are not actively sought after by collectors or research scientists. A taxonomic group with wide scientific and public popularity, such as dinosaurs, may act as ‘trophy specimens’ [44], with high collection interest and increased likelihood of attaining funding for international fieldwork resulting in higher rates of parachute science. In contrast, collection of Palaeozoic echinoids appears to have mostly taken place opportunistically during national geological surveys in the late nineteenth and early twentieth centuries.

These data show that unpublished museum specimens exhibit the same biases as observed within publications in the PBDB; as such, it is likely that the conclusions from Raja *et al*. [12] can be extrapolated to the wider available fossil record. This illustrates that even with a shift towards ‘big data’ in palaeontology through the digitization and integration of unpublished museum specimens [15], underlying structural issues will continue influencing our understanding of deep-time biodiversity. The impacts of scientific colonialism [45] and other geological, geographical, historical and socioeconomic factors will continue to contribute towards spatial heterogeneity in the availability of the fossil record, which will in turn profoundly influence palaeobiological analyses [8,12,46]. The digitization and integration of museum collections with currently available records additionally has the potential to aid in this process, providing an opportunity to consider historical inequalities and injustices [47,48]. Pathways integrating museum specimens within globally accessible datasets must actively engage with this historical context, ensuring that lower-income countries are equally supported with regards to collection, storage and digitization of fossil collections, and such issues can only be equitably addressed through ethical and sustainable collaborations, funding and data management going forwards [12,48,49].

It has become increasingly apparent that spatial data absence caused by heterogeneity in sampling skews palaeobiogeographic patterns [9,11,50]. Our results clearly show that the inclusion of museum ‘dark data’ contributes significant unique information to various spatial patterns observed in the fossil record; global occupancy, latitudinal range and geographic range size of genera are increased between 28−35% on average with the addition of museum only occurrences, which could have ramifications on other fields of study. Range size is seen as an important metric in species conservation due to acting as a key indicator of extinction selectivity and risk in both extinct [51,52] and extant [53,54] systems. While recent simulation work has found that range size can be adequately reconstructed from the fossil record [55,56], our results indicate that using only data from the published record may result in these figures being underestimated. This may in part be due to the increased preservation potential within the marine realm requiring a larger sample size to accurately reflect ranges [55] and indicates that inclusion of museum data may have significant impacts on reconstructing variations in biogeographic range size through time.

Conversely, our results indicate that other patterns show limited impact from the addition of ‘dark data’ occurrences. The temporal range of genera is only shown to increase by 5% on average when these specimens are incorporated; this is potentially due to differing researcher aims. Individual museum collections are predominantly focused on specific localities or time intervals based on historical links or researcher interests and are unlikely to have been collected with the aim of increasing temporal coverage for individual taxa. Conversely, occurrences that enter the published record are more likely to be aimed at highlighting unique data, such as the oldest or youngest appearance of a particular taxon, which can expand the temporal range of groups and also play an important part in dating the age of extant clades [57]. This effect can potentially be identified in the distribution of occurrences within their total temporal range (figure 4B), where ‘dark data’ occurrences appear to be relatively ‘bottom weighted’ within the total age range and more unequally distributed than those in the published record. While this limits the impact of ‘dark data’ on directly perceived temporal patterns, other analyses relying on the distribution of occurrences throughout time to estimate sampling rates, such as the fossilized birth–death process [58], PyRate [59] or *cal3* [60] might be more significantly impacted by these differences in distribution.

Our results also indicate that the addition of museum ‘dark data’ has the capacity to impact analyses and in turn alter interpretations regarding the evolutionary histories of groups. When ‘dark data’ are included in the calculation of coverage-based rarefied genus diversity through time, we see slight, but important differences in observed diversity. In particular, there is a small Devonian peak which is absent from the published diversity curve (dataset B), but present in the complete dataset (dataset D). The Devonian has been implicated as a particularly important interval of time for understanding echinoid evolution, as there is likely faunal turnover at the family level associated with late Devonian extinctions [61]. Thus ‘dark data’ may be providing additional important information on this interval which is absent from solely the published record. More significantly however, we also find that the covariates included within the best-fitting model using a global dataset change significantly with the addition of ‘dark data’; additional covariates included in the best-fitting model for the published record are removed, with only collections showing a statistically significant control on diversity within Palaeozoic echinoids. Thus, we shift from a case where environmental drivers are found to best predict changes in biodiversity, to one where these changes are simply the result of differential sampling. Broad consistency between raw and coverage-based rarefaction estimated diversity may suggest that the global Palaeozoic record of echinoids is therefore controlled by sampling intensity, rather than genuinely biological patterns, an interpretation that would not have been made without the inclusion of ‘dark data’. These results indicate that the exclusion of ‘dark data’ from broader scale palaeontological analyses can seriously influence results and their interpretations, and may be detrimental to understanding drivers of macroecological change in deep-time.

Other analyses show more limited impacts. While there are subtle differences between analyses as described above, both coverage-based rarefaction and SRA results show a broad degree of consistency between the complete dataset, the published record, and museum-only data. This is similar to the findings of Davis & Pyenson [44], who found that while abundance data showed distinct differences between the published record and museum collections, estimates of diversity remained consistent. This is somewhat expected, due to a central aim of palaeontological publications being to produce a unique account of taxa that existed throughout both time and space [44]. Consequently, an understanding of certain broad palaeobiological patterns can likely be ascertained with currently available datasets lacking the inclusion of unpublished museum specimens. In contrast, previous work found that invertebrate diversity in the Western Interior Seaway of North America was underestimated by factor of between 3 and 5, depending on the taxonomic group, when comparing field and museum specimens to the published record [14]. This discrepancy could be due to the differences in geographic and temporal sampling intensity. As discussed above, the Palaeozoic echinoid record is both relatively sparse and reasonably well sampled for what is currently available. In contrast, the Western Interior Seaway has an extensive geological record, with abundant remains of a variety of shelly marine invertebrates throughout its duration [62]. Consequently, proportionally more data may have been published in the echinoid fossil record, improving sample accuracy [44]. It is therefore possible that published data can be considered a more accurate representation of the current sum of scientific knowledge for a taxonomic group in cases of reduced overall richness and availability in the fossil record, yet high research interest (e.g. terrestrial vertebrate clades [63]). This serves as an important reminder to incorporate geographic, temporal, taxonomic and taphonomic differences when interpreting palaeobiological analyses. Similarly, the increase in unique geographic locations with the addition of ‘dark data’ in the present contribution is noticeably lower than the increase in location numbers found in Marshall *et al*. [16], which estimated that unpublished museum records contained 23 times more locations than those shown in the PBDB. This is likely to be due to the relative rarity of Palaeozoic echinoids in comparison to the broad taxonomic bracket of their study (fossil invertebrates). Conversely, as echinoids become more abundant during the Carboniferous and preserved occurrences are no longer restricted to more Lagerstätten-like deposits [19], we see a shift to ‘dark data’ providing a wider array of unique spatial data (electronic supplementary material, figure S4*b*). The increased presence of widespread carbonate ramps during the Mississippian resulted in an abundance peak for crinoids and consequently has been a focus of collection for crinoid workers [64]. Echinoids have often ended up as ‘by-catch’ during collection from this interval; this increase in unique spatial data might consequently be a product of more specimens being collected and entering museums but not entering the published record due to them not being the primary focus of the workers collecting them. This shows how broader shifts in the dominance of certain lithologies over the Phanerozoic [65] may have additional unseen effects for the sampling of fauna. It is therefore possible that the impact of museum collections is likely to depend on the abundance of the group or taxon of study due to both biological and preservational conditions, and that geographic measures for other groups, particularly invertebrates, may see an even greater impact from the inclusion of museum ‘dark data’.

It should also be noted that in comparison with the museum only and published record datasets, the PBDB dataset did not mirror the patterns observed within the complete dataset in either coverage-based rarefaction or SRA results. Echinoids receive little attention from palaeontologists in comparison to other invertebrate groups within the Palaeozoic [37], and consequently their record within the PBDB is not an accurate representation of their published record. As such, they are likely to represent an analogue to other taxonomic groups that are similarly underrepresented within studies of the fossil record. While the PBDB remains an invaluable resource for all palaeontologists, we urge caution in assuming consistency in data completeness between different taxonomic groups and time periods. We strongly encourage echinoid researchers, and workers for other underrepresented groups, to join efforts to make the PBDB more reflective of the available published data.

Our results indicate that the integration of museum ‘dark data’ has the capacity to impact the results of macroevolutionary analyses using palaeontological occurrence data. However, it is important to consider that museum collections are also not perfect representations of the available fossil record [66]. Previous studies examining differences between museum collections or their proxies and field collection surveys have found biases relating to collector selection interest and abundance accuracy that have rendered various analyses of community assemblage inaccurate [40,44,46,67,68]. Further work across a broader selection of taxonomic groups is necessary to more fully understand how these collection biases transfer from the field, to museum collections, and then finally to datasets used for palaeobiological analyses.

## 5. Conclusions

Accurately estimating and interpreting past biodiversity and biogeographic patterns is fundamental for understanding broader macroevolutionary trends and for informing predictions of future species distributions necessary for conservation efforts [69,70]. Our results indicate that the inclusion of unpublished museum specimens has a strong impact on perceived biogeographic patterns and can dramatically alter interpretations of macroevolutionary analyses carried out on specific taxonomic groups. As such, the inclusion of ‘dark data’ in such analyses is essential for obtaining a complete understanding of deep-time macroevolutionary patterns. We consequently recommend that studies using palaeontological occurrence datasets, particularly ones assessing biogeographic patterns or running modelling approaches, attempt to incorporate unpublished museum specimens into their analyses or express caution in their interpretations if this is not feasible. In contrast, studies assessing trends in diversity are less likely to benefit from the inclusion of unpublished museum specimens. Our study demonstrates the importance of curation and care of museum specimens in the twenty-first century [15] and the necessity of efforts to digitize and incorporate museum collections into broader online databases, such that their unique data can be appropriately leveraged for understanding both ancient and future patterns of biodiversity.

## Data Availability

All datasets and R scripts to run analyses are available on Dryad [21]. Supplementary material is available online [71]: ESM 1: Supplementary figures and dataset information. ESM 2: Supplementary tables and datasets.
